# Long-term follow-up results of percutaneous closure of atrial septal defect with a novel biodegradable poly-L-lactic acid device in pediatrics: data from a prospective, single-center trial

**DOI:** 10.3389/fcvm.2026.1804642

**Published:** 2026-04-20

**Authors:** Yi-Fan Li, Zhao-Feng Xie, Bo-Ning Li, Jun-Jun Shen, Shu-Shui Wang, Yu-Mei Xie, Zhi-Wei Zhang

**Affiliations:** 1 Department of Pediatric Cardiology, Guangdong Cardiovascular Institute, Guangdong Provincial People’s Hospital (Guangdong Academy of Medical Sciences), Southern Medical University, Guangdong Provincial Key Laboratory of South China Structural Heart Disease, Guangzhou, China; 2 Department of Pediatric Cardiology, Shenzhen Children’s Hospital, Shenzhen, China

**Keywords:** atrial septal defect (ASD) closure, biodegradable, clinical trial, device, pediatric

## Abstract

**Background:**

The poly-L-lactic acid (PLLA) occluder is a novel, fully biodegradable device designed for percutaneous atrial septal defect (ASD) closure. First-in-human studies have demonstrated its preliminary safety and efficacy.

**Objective:**

This study aimed to evaluate the 5-year safety and efficacy of the PLLA device for percutaneous ASD closure in a single-center pediatric cohort.

**Methods:**

From May 2018 to August 2019, 36 patients with clinically significant ASD were enrolled and underwent percutaneous closure using the PLLA device. Follow-up assessments were conducted at discharge and at 1, 3, 6, 12, 24, 36, 48, and 60 months post-implantation. The primary endpoint was a composite of clinical success, defined as successful closure and absence of major complications at the 60-month follow-up.

**Results:**

Successful device implantation was achieved in 35 of 36 patients (97.2%). All of the 35 patients completed the 5-year follow-up. The closure success rate and complete closure rate at 5 years were 85.7% (30/35) and 77.1% (27/35), respectively. Clinically significant residual leaks at 5- year visit were observed in 5 patients (14.3%). A total of 5 complications (14.3%) occurred, consisting of cardiac arrhythmia (*n* = 3), moderate mitral regurgitation (*n* = 1), and migraine (*n* = 1). Patients with a larger baseline ASD indexed diameter (>15.18 mm/m^2^) and a smaller device-to-defect ratio (<1.47) showed an increased risk of residual leaks after PLLA device implantation.

**Conclusion:**

Long-term follow-up confirms a favorable safety profile for percutaneous ASD closure using the PLLA device, with a cumulative complication rate of 14.2% and no major adverse events reported over 5 years. However, the incidence of residual leaks remains noteworthy, suggesting suboptimal long-term efficacy in pediatric patients and in cases involving large defects.

## 1 Introduction

Percutaneous device closure has become the preferred method over surgical repair for secundum ASD with suitable anatomy. Currently, the available closure devices consist of a metallic framework and a synthetic fabric which provide a permanent septal occlusion through a combination of mechanical closure and fibrous encapsulation of the device. Potential complications following implantation of permanent devices, such as cardiac erosion or perforation, thrombus formation, frame fractures, and nickel allergy have been described (1–3). Furthermore, these metallic implants will obstruct a potential transseptal access to the left atrium for future treatment of acquired heart disease. Therefore, new strategies for ASD closure with a biodegradable device have been explored. Some of these devices have been tested in clinical trials, such as BioSTAR occluder and Carag bioresorbable septal occlude (CBSO), but neither of them is total biodegradable (4–7).

The PLLA device is a novel total biodegradable device specifically designed for the closure of ASD. It consists of a PLLA framework and three PLLA membranes which are sewn into two umbrella-shaped disks and the waist (8, 9). This configuration is intended to promote endothelialization and neo-tissue coverage, thereby enhancing the closure efficacy. Previous preliminary studies involving five pediatric cases have indicated the initial safety and efficacy of this device (10). In this study, we further evaluate the long-term efficacy and safety of percutaneous ASD closure using the PLLA device over a 60-month period in a single-center pediatric cohort.

## 2 Materials and method

### 2.1 Study design

From May 2018 to August 2019, pediatric patients aged 3 to 18 years with a body weight of at least 10 kg, who were scheduled for percutaneous ASD closure, were screened for eligibility at our center. The study protocol was approved by the Institutional Review Board of Guangdong Provincial People's Hospital [Approval No. (2018) 20-2] and was conducted in accordance with the principles of the Declaration of Helsinki. Written informed consent was obtained from all patients and their legal representatives. This study forms part of a prospective, multi-center trial evaluating the PLLA device for the treatment of secundum ASD (ClinicalTrials.gov, Identifier: NCT03601039).

### 2.2 Patient selection

Inclusion criteria were a secundum ASD of ≥5 mm and ≤30 mm measured by transthoracic echocardiography (TTE) or transesophageal echocardiography (TEE), a left-to-right shunt with Qp/Qs ≥ 1.5:1 or presence of right ventricular volume overload, the presence of a distance of >7 mm from the margins of the ASD to the atrioventricular valves, >5 mm to the right upper pulmonary vein and inferior/ superior vena cava as measured by TTE/TEE. Patients with concomitant congenital heart defects or acquired heart disease which could contribute to additional intervention during the study period were excluded from the study.

### 2.3 Study device

**Figure 1:**
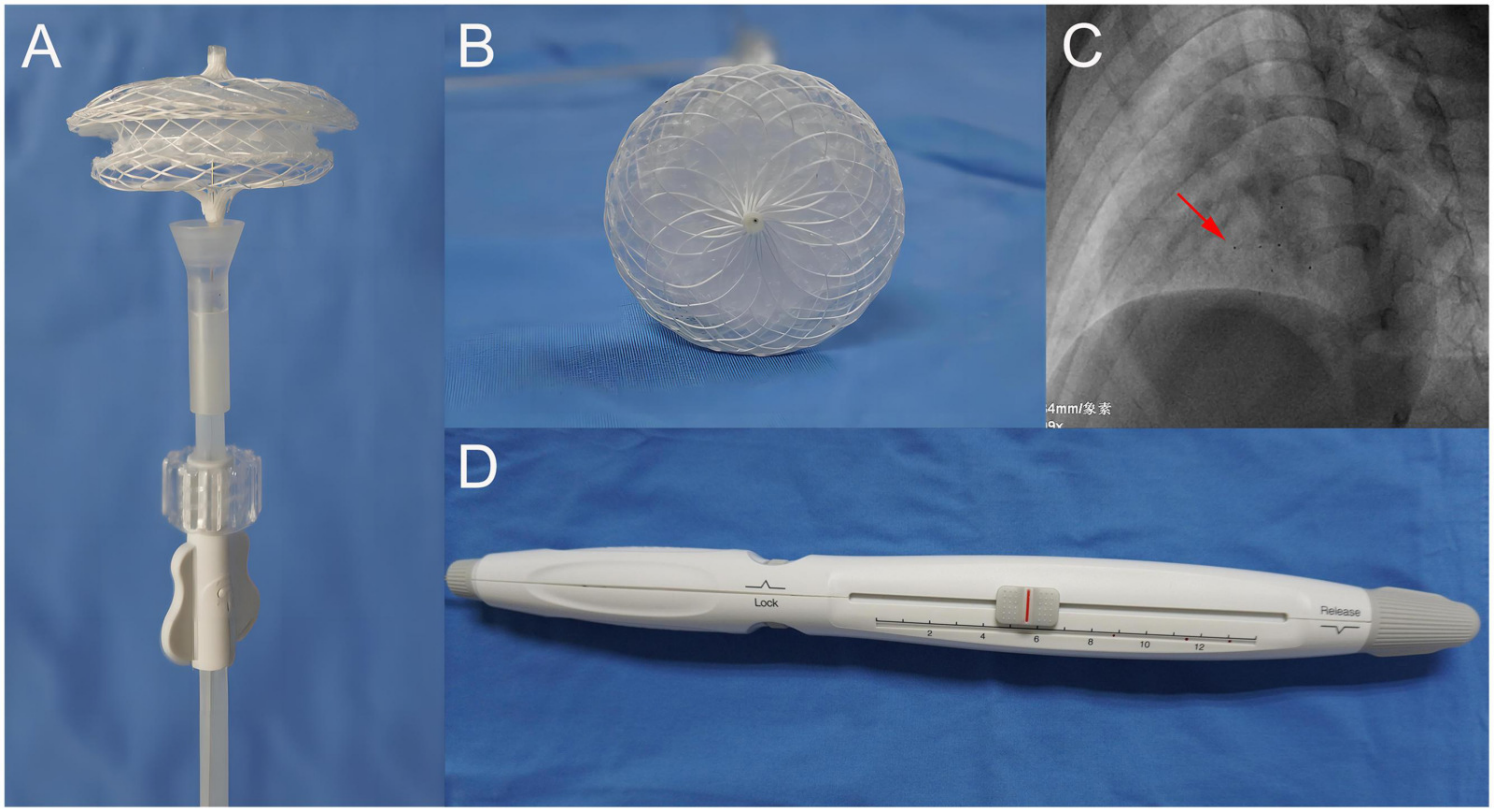
The PLLA device. **(A)** The “unbuttoned” state of the device. **(B)** Frontal view of the device. **(C)** Fluoroscopic views of the device demonstrating enhanced visibility provided by the 7 platinum-iridium markers (red arrow). **(D)** The handle of the delivery system. PLLA, poly-L-lactic acid.

The PLLA device is composed of a double-disc PLLA framework and three pieces of PLLA membrane mounted on the two discs and the waist (Figures 1A,B). Seven platinum-iridium markers were added to facilitate the x-ray visibility (Figure 1C). The occluder is fixed by a PLLA locking piece that passes through the center of the device from the left to the right atrial disc, and connects to the delivery system (11). The device can be positioned and retrieved at an “unbuttoned” state, and can be deployed at a “buttoned” state by controlling the handle on the delivery system (Figure 1D). The PLLA device is available in waist sizes from 6 to 32 mm at 2-mm increment deliverable through 8-14 Fr sheaths.

### 2.4 Study procedure

The closure procedure was conducted under general anesthesia, with venous access established via the right femoral vein. Diagnostic catheterization was performed for hemodynamic assessment. During the procedure, the size and anatomy of the ASDs were evaluated using TTE/TEE. The recommended device size was 4–8 mm larger than the measured defect diameter: for small to moderate ASDs with stiff, firm rims, an oversizing of 4–6 mm was selected; whereas for large ASDs with floppy or thin rims, an oversizing of 6–8 mm was applied. The delivery and deployment of the PLLA device followed methods detailed in our prior publications (10). In brief, the device was advanced into the left upper pulmonary vein through a delivery sheath under fluoroscopic guidance. The sheath was then withdrawn to the right atrium, allowing full device exposure within the atrium. Under fluoroscopic and echocardiographic guidance, the left atrial disc, waist, and part of the right atrial disc were sequentially deployed to accommodate the defect. The left disc was gently retracted against the atrial septum, after which the right atrial disc was fully expanded under echocardiographic guidance with constant tension applied to the delivery cable. Device position and adjacent structures were assessed by echocardiography to rule out residual leaks, atrioventricular valve interference, or venous obstruction. Upon confirmation of optimal positioning, the delivery system was activated to release the device. Following deployment, the device position was verified using the seven radiographic markers visible on fluoroscopy. A final TTE/TEE was then performed to assess the device profile and to evaluate any residual leaks. The above steps are demonstrated in Supplementary Material Videos S1–S4.

### 2.5 Follow-up evaluation

Within 24 h post-closure, all patients underwent TTE and ECG to confirm appropriate device position and exclude device embolization, residual leaks, interference with adjacent structures, pericardial effusion, and arrhythmia. These assessments were repeated at 1, 3, 6, 12, 36, 48, and 60 months following the procedure. Follow-up echocardiographic evaluations were conducted by experienced cardiologists at our institution. All measurements were initially reviewed and verified by two independent experts blinded to clinical data. Any discrepancies were resolved through consensus or adjudication by a third expert. Echocardiographic data were subsequently transferred to and independently analyzed by a centralized core laboratory at Guangdong Cardiovascular Institute, which remained blinded to patient allocation and clinical information. Chest x-ray was performed at 24 h, 6 months, and 12 months to assess for potential device embolization. All patients received aspirin (3–5 mg/kg once daily) for six months post-implantation.

### 2.6 Outcome measures

The primary endpoint was composite clinical success, comprised of closure success and safety at the 60-month follow-up evaluation. The criteria for clinical success: (1) closure success with either complete occlusion or clinically insignificant leak; (2) the absence of major complications through the 60-month follow-up. Secondary end points included efficacy and safety evaluation at each of the follow-up visits.

Efficacy outcome included assessment of defect evaluated by residual leak at each of the follow-up visits by TTE. Residual leak was categorized as: (1) complete occlusion; (2) a clinically insignificant leak was defined as a residual leak ≤ 2.0 mm; (3) a clinically significant leak was defined as a residual leak >2.0 mm. Residual leak determined by color-doppler TTE was graded as mild (≤2.0 mm), moderate (2.1–4.0 mm), and large(> 4.0 mm) (12).

Safety outcome included assessment of all device-related and procedure-related complications through the 60-month follow-up. Major complications were defined as events related to the device or the procedure that result in prolonged hospital stay, readmission to the hospital or permanent damage (13). These included device embolization, cardiac tamponade, endocarditis, perforation of cardiovascular structures by the device or delivery system, new cardiac arrhythmia requiring major treatment, a thrombotic/thromboembolic event resulting in clinical sequelae, and repeat procedure to the target defect (3).

### 2.7 Statistical analysis

All statistical analyses are conducted using SPSS Statistics, version 23 (IBM Corp., Armonk,281 N.Y., USA). Continuous variables with normal distribution are expressed as mean ± SD and median (minimum, maximum) for those that were not normally distributed. For comparison between groups, a student's T-test for normally distributed continuous data or Wilcoxon rank-sum test for non-normally distributed continuous data were performed. Chi-square test or Fisher exact test were performed for categorical variables. Statistical significance was claimed if a 2-sided test obtained a *p* value of <0.05.

## 3 Results

### 3.1 Patient characteristics and procedural outcome

38 pediatric patients were screened echocardiographically, and a total of 36 patients met the criteria and were enrolled in the study. The median age was 5.7 years (range 3.1–17.9 years), and the median weight was 18.25 Kg (range, 10–56 Kg). Both fluoroscopic and TTE/TEE guidance were employed during the procedure. TEE was used in 2 patients, while TTE was used in the remaining 34 patients. Technical success was achieved in 35 (97.2%) of the 36 patients with delivery attempts. The pre-procedural baseline median ASD diameter was 10.3 mm (range, 5.4–24.0 mm), and the median ASD indexed diameter (ASD/body surface area) was 12.25 mm/m^2^ (range, 5.85–31.41 mm/m^2^). The median procedure time was 59.5 min (range, 33–101 min), and the median fluoroscopy time of 7.4 min (range, 3.4–14.3 min). Further patient demographics and procedure details are detailed in Table 1.

**Table 1:** Baseline characteristics of patients and procedural data.

No. of patients who underwent implantation	*n*=36
Sex	
Male, *n* (%)	15 (41.7)
Female, *n* (%)	21 (58.3)
Patient age, yrs	
Mean(SD)	7.12 (4.32)
Median	5.7
Range(min, max)	3.1–17.9
Age distribution, yrs	
Child (3–11), n(%)	31 (86.1)
Adolescent (12–17), n(%)	5 (13.9)
Weight, Kg	
Mean(SD)	25.10 (14.97)
Median	18.25
Range(min, max)	10–56
Body surface area, m^2^	
Mean(SD)	0.89 (0.35)
Median	0.74
Range(min, max)	0.49–1.79
Baseline ASD diameter by TTE/TEE, mm	
Mean(SD)	11.54 (4.58)
Median	10.3
Range(min, max)	5.4–24.0
Baseline ASD indexed diameter(ASD/body surface area), mm/m^2^	
Mean(SD)	13.68 (5.50)
Median	12.25
Range(min, max)	5.85–30.41
ASD characteristics	
Multifenestrated	4 (7.7%)
Atrial septal aneurysm	7 (13.5%)
Qp/Qs	
Median	1.51
Range(min, max)	1.1–3.6
PLLA device size, mm	
Median	16
Range(min, max)	10–32
Device-to-defect ratio	
Median	1.58
Range(min, max)	1.2–2.4

ASD, atiral septal defect; TTE, transthoracic echocardiography; TEE, transesophageal echocardiography; PLLA, poly-L-lactic acid.

Device implantation was unsuccessful in 1 patient (2.8%). In this patient (Participant No.36, 17.9 years old, female) with a 24 mm ASD, a 32-mm PLLA device was initially deployed. The device prolapsed into the right atrium despite several deployment attempts, possibly attributed to the device being undersized for the defect. The PLLA device was then replaced by a 34-mm nitinol ASD occluder. No major periprocedural complications related to the device or delivery system occurred, and none of the procedural complications resulted in long-term sequelae.

### 3.2 Follow-up results

#### 3.2.1 Closure success

**Figure 2:**
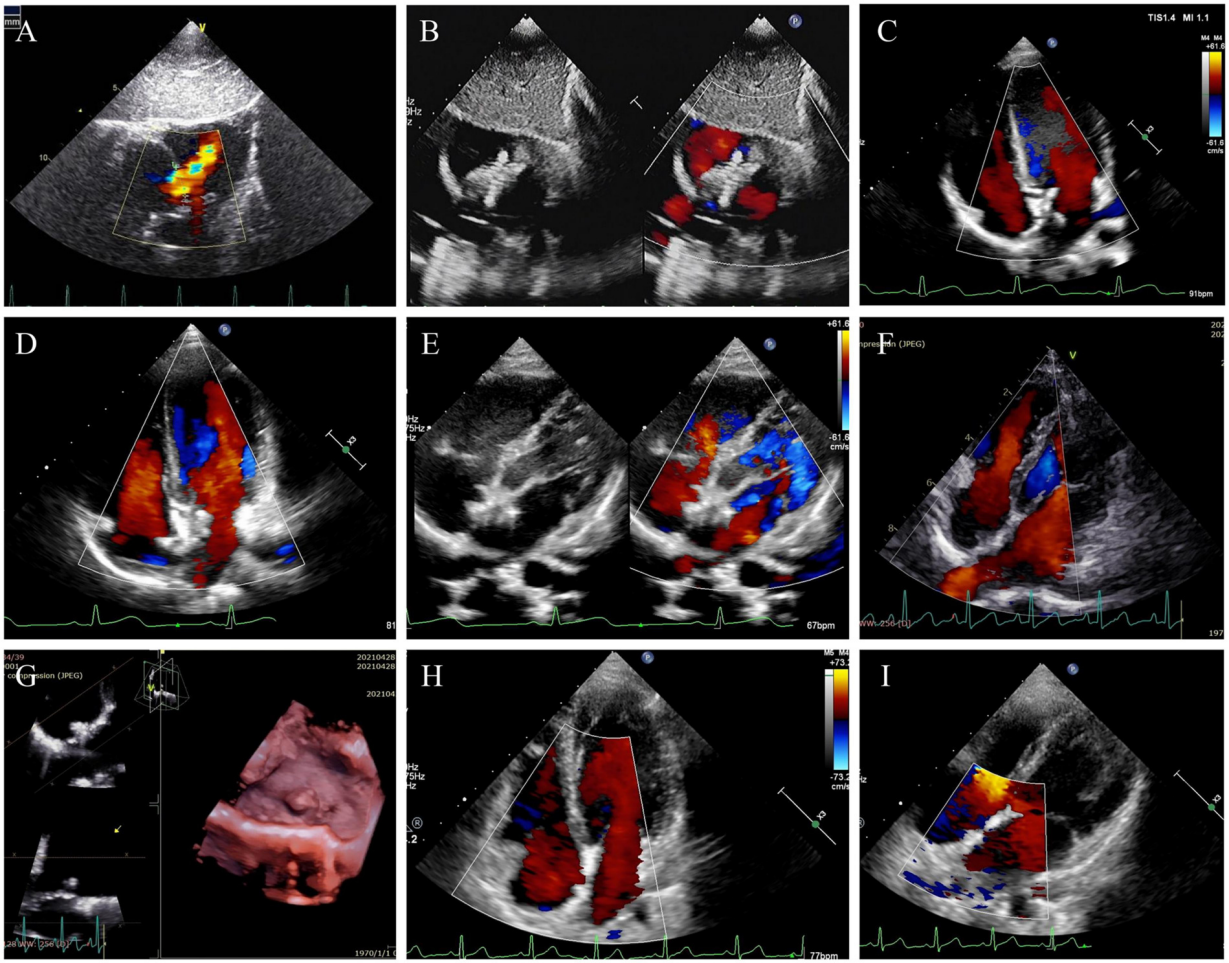
TTE follow-up results through the 5-year visits illustrating the degradation process of the PLLA device in participant No.3 (male, 6Y6M, 21 kg). **(A)** Pre-procedural TTE revealed a 15-mm ASD. **(B)** A 20-mm PLLA device was implanted, with no residual shunt detected at 24 h post-implantation. **(C)** At the 6-month follow-up, morphological changes in the device were observed, suggesting the onset of degradation. **(D)** At 1 year, TTE revealed a reduction in device size compared with the 6-month assessment. **(E)** At 2 years, partial degradation of the device was evident. **(F)** At 3 years, the majority of the device had undergone degradation. **(G)** 3D-TTE at the 3-year follow-up confirmed near-complete degradation of the device disc, with only the right atrial screw remaining visible. **(H,I)** At 4- and 5-year follow-ups, complete device degradation was observed, and the atrial septum appeared intact and covered by neo-tissues.

A total of 35 patients completed the 60-month follow-up assessment. Successful defect closure was achieved in 34 patients (97.1%) at 6 months, 34 (97.1%) at 12 months, 32 (91.4%) at 24 months, 29 (82.9%) at 36 months, and 30 (85.7%) at 60 months (Table 2). Follow-up TTE demonstrated that the two PLLA discs began to degrade within 1 year, with the degradation process accelerating between 1 and 2 years. By 3 years, the majority of both discs had resorbed, with the exception of the screw on the right disc. At the 5-year evaluation, both PLLA discs had undergone complete degradation, and the atrial septum had been fully reconstructed with newly formed autologous tissue (Figure 2).

**Table 2:** Residual leaks and clinical significance.

Patients with successful implantation (*n* = 35)	30 days	6 months	12 months	24 months	36 months	48 months	60 months
Clinical closure success	35/35 (100)	34/35 (97.1)	34/35 (97.1)	32/35 (91.4)	29/35 (82.9)	29/35 (82.9)	30/35 (85.7)
Complete occlusion	27/35 (77.1)	30/35 (85.7)	31/35 (88.6)	28/35 (80)	28/35 (80)	27/35 (77.1)	27/35 (77.1)
Clinically insignificant leak	8/35 (22.9)	4/35 (11.4)	3/35 (8.6)	4/35 (11.4)	1/35 (2.9)	2/35 (5.7)	3/35 (8.6)
Clinically significant leak	0/35 (0)	1/35 (2.9)	1/35 (2.9)	3/35 (8.6)	6/35 (17.1)	6/35 (17.1)	5/35 (14.3)
Measured closure success	35/35 (100)	34/35 (97.1)	34/35 (97.1)	32/35 (91.4)	29/35 (82.9)	29/35 (82.9)	30/35 (85.7)
Complete occlusion	27/50 (77.1)	30/35 (85.7)	31/35 (88.6)	28/35 (80)	28/35 (80)	27/35 (77.1)	27/35 (77.1)
0–2.0 mm residual leak	8/35 (22.9)	4/35 (11.4)	3/35 (8.6)	4/35 (11.4)	1/35 (2.9)	2/35 (5.7)	3/35 (8.6)
Measured closure failure	0/35 (0)	1/35 (2.9)	1/35 (2.9)	3/35 (8.6)	6/35 (17.1)	6/35 (17.1)	5/35 (14.3)
2.1–4.0 mm residual leak	0/35 (0)	1/35 (2.9)	1/35 (2.9)	3/35 (8.6)	4/35 (11.4)	3/35 (8.6)	3/35 8.6)
>4.0 mm residual leak	0 (0)	0 (0)	0 (0)	0 (0)	2/35 (5.7)	3/35 (8.6)	2/35 (5.7)

Values are *n* (%).

**Figure 3:**
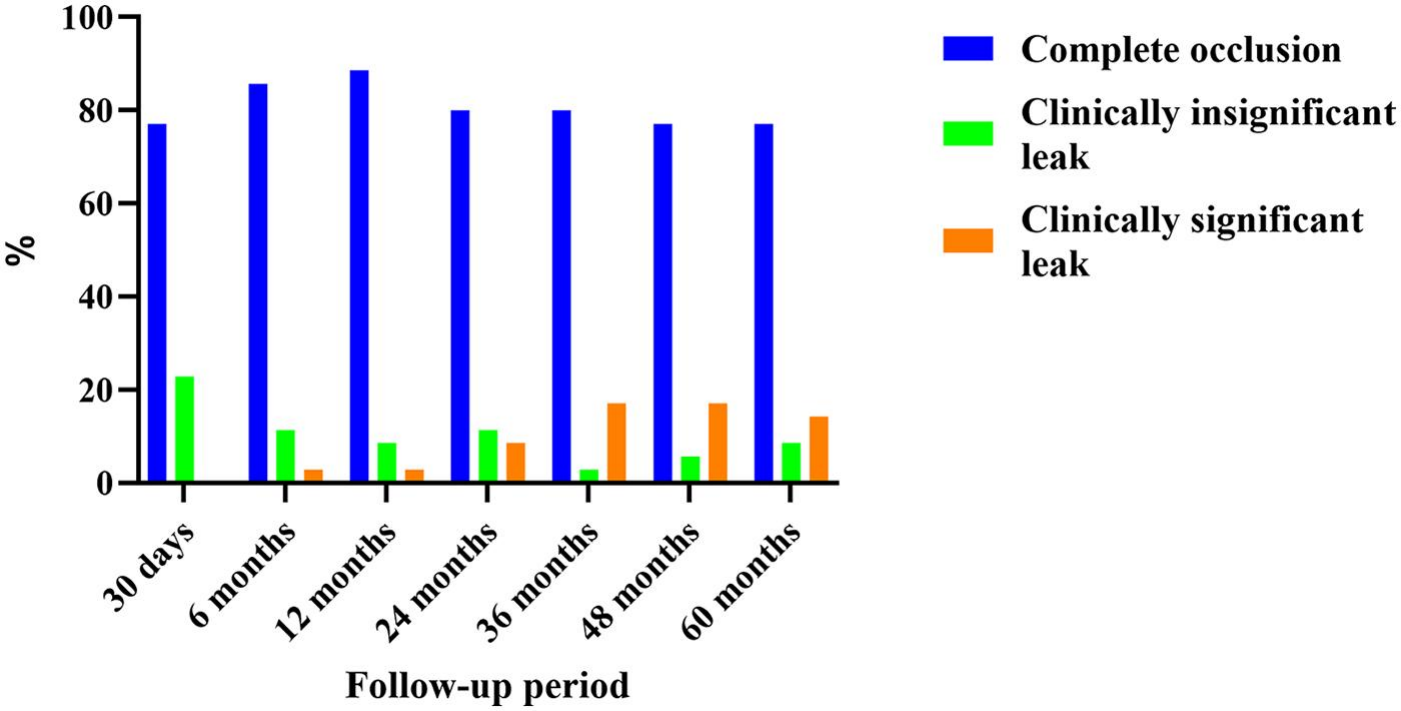
Residual leak status demonstrated by TTE at different follow-up period. TTE, transthoracic echocardiography.

Complete closure of the defect was achieved in 30(85.7%) patients at 6 months, 31(88.6%) patients at 12 months, 28(80%) patients at 2 years, 28(80%) patients at 3 years, 27(77.1%) patients at 4 years, and 27(77.1%) patients at 5 years. The incidence of residual leaks increased progressively from the second year through the fourth and fifth years of follow-up, with the proportion of significant residual leaks reaching its peak at the four-year visit. Detailed information on residual leaks is provided in Table 2 and Figure 3.

**Figure 4:**
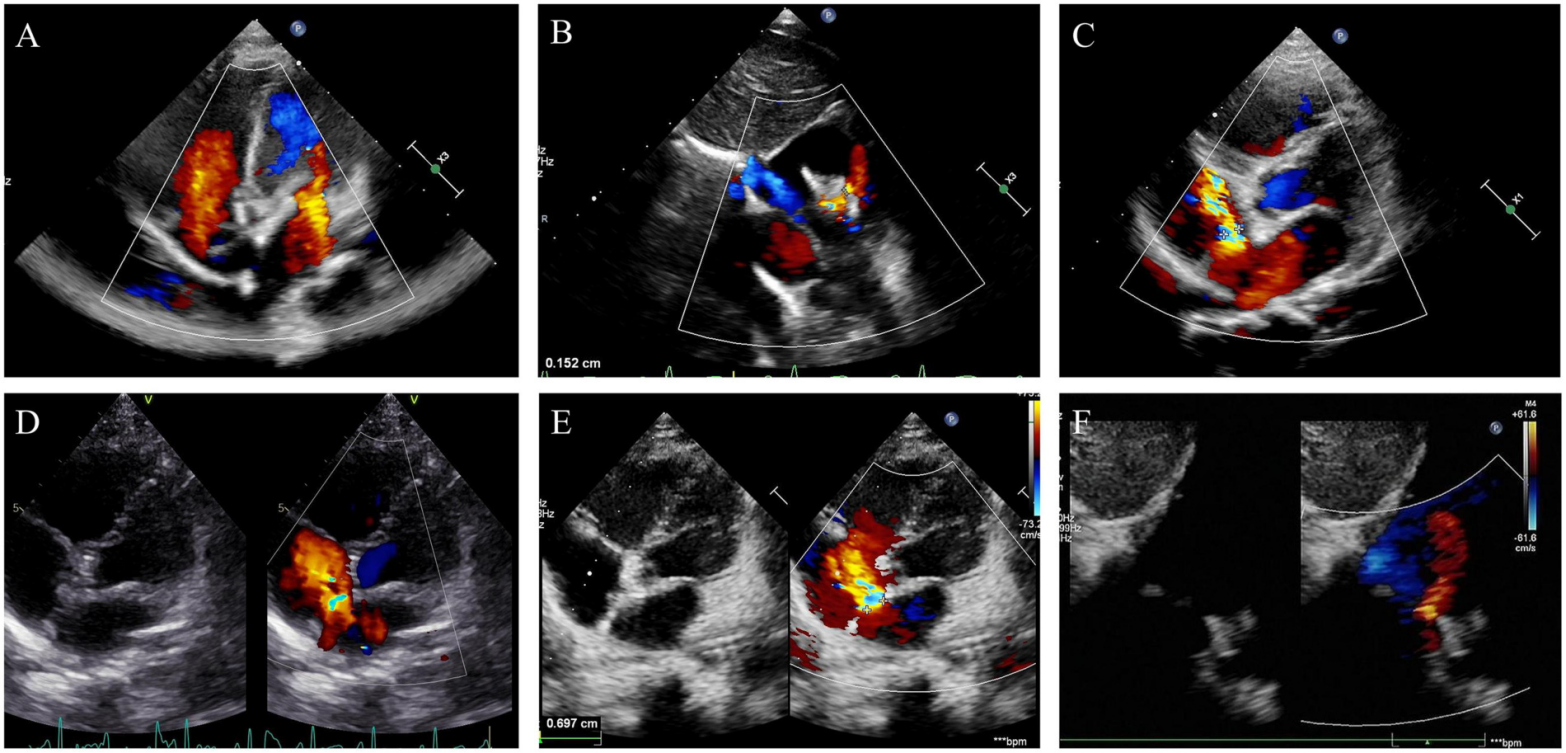
TTE follow-up results demonstrating progressive residual leaks after PLLA device implantation in participant No.2 (male, 3 years old male, 10 kg). **(A)** 6 month follow-up showed complete occlusion with no residual leak. **(B)** Small residual leak first detected at the 1-year follow-up. Reproduced from “The development of residual shunt in Patient no. 2. (a-b) A mild residual shunt of 1 mm was detected at the 12-month visit” by Yifan Li, Yumei Xie, Boning Li, Zhaofeng Xie, Junjun Shen, Shushui Wang and Zhiwei Zhang, licensed under CC BY 4.0. **(C)** Progression to a large residual leak (4.4 mm) at the 2-year follow-up. **(D)** Further enlargement of the residual defect observed at the 3-year follow-up. Reproduced from “The development of residual shunt in Patient no. 2. (e-f) a large residual shunt of 4.3 mm was detected at the 36-month visit. The device was mostly degraded” by Yifan Li, Yumei Xie, Boning Li, Zhaofeng Xie, Junjun Shen, Shushui Wang and Zhiwei Zhang, licensed under CC BY 4.0. **(E,F)** Follow-up at 4 and 5 years, respectively, revealing a persistent residual leak measuring 6-7 mm. The residual defect was consistently located at the anterosuperior aspect of the atrial septum.

5 patients (14.3%) were reported to have clinically significant leaks at 5-year follow-up. Among them, 3 experienced moderate residual leaks, and 2 experienced large residual leaks. No patients required further intervention as yet. All of the residual leaks were located at the anterosuperior site of the atrial septum, close to the anterior-superior aortic rim of the defects (Figure 4).

Receiver operating characteristic (ROC) curve analysis was conducted to identify predictors of residual leaks of all degree, based on patient demographics and defect characteristics. Among the variables assessed, the baseline ASD indexed diameter and the device-to-defect ratio emerged as the most predictive parameters, with area under the curve values of 0.926 [95% confidence interval (CI): 0.838–1.014; *p* < 0.001] and 0.801 (95% CI: 0.644–0.958; *p* = 0.011), respectively (Supplementary Figures S1 and S2). For the ASD indexed diameter, the optimal cutoff value for predicting a significantly increased risk of residual leak was >15.18 mm/m^2^, which yielded a sensitivity of 100% and a specificity of 85.2%. For the device-to-defect ratio, the optimal cutoff value was <1.47, corresponding to a sensitivity of 75% and a specificity of 85.2%.

#### 3.2.2 Complications

Of the 35 patients who completed 5-year follow-up, no major complications were reported. 5 minor complications (14.3%) occurred, consisting of cardiac arrhythmia (*n* = 3), moderate mitral regurgitation (*n* = 1), and migraine (*n* = 1). The primary ECG abnormalities observed throughout the study period included frequent atrial premature contractions, frequent ventricular premature contractions, first-degree atrioventricular block, and paroxysmal atrial tachycardia.

Of the three patients (No. 35, 21, and 12) who developed cardiac arrhythmia, Participant No. 35 exhibited frequent atrial premature contractions within 24 h post-procedure, which resolved spontaneously without intervention. The remaining two participants (No. 21 and 12) presented with new-onset arrhythmia during follow-up. Participant No. 21 (male, 8 years old, 53 kg) presented with frequent ventricular premature contractions and first-degree atrioventricular block (I°AVB) at the 4-year visit. While anti-arrhythmic medication resolved the premature contractions, I°AVB recurred at the 5-year follow-up. Participant No.12(female, 11 years old, 29 kg) reported recurrent palpitations at the 5-year visit, which were diagnosed as paroxysmal atrial tachycardia and managed with beta-blockers.

Participant No.30 (female, 10 years old, 31.5 kg) presented with new-onset moderate mitral regurgitation (MR) within one month following implantation of a 30-mm PLLA device for a 20-mm ASD. TTE revealed contact between the edge of the occluder disc and the anterior mitral valve leaflet. The patient remained asymptomatic with stable MR on serial follow-up, and no re-intervention was required.

Participant No.31 (female, 6 years old, 18 kg) reported new-onset migraine within the first month following device closure. TTE showed no thrombus formation on the device and no residual shunt. There were no cerebral thromboembolic events documented. Although a definitive causal relationship could not be established, the migraine was considered potentially related to the device implantation. The migraine symptoms gradually improved after the first month and resolved completely during later follow-up assessments.

## 4 Discussion

So far, reported long-term experience with a biodegradable septal device in humans is limited. In recent years, substantial efforts have been dedicated to the development of fully biodegradable occluders. These devices are fabricated from polymeric materials such as PDO and PLLA, which confer greater softness compared to traditional nitinol occluders, potentially reducing the risk of cardiac tissue injury. A previous study has demonstrated that the use of a biodegradable ventricular septal defect occluder was associated with a significantly lower incidence of sustained conduction block than that observed with a metallic occluder (14). Furthermore, biodegradable occluders primarily serve as a temporary scaffold to guide autologous tissue repair and are eventually completely absorbed, leaving no metallic residue *in situ*. This characteristic may help mitigate long-term complications associated with the permanent implantation of metal alloys in cardiac tissue.

To our knowledge, this is the first study reporting the long-term safety and efficacy of a total biodegradable device for ASD closure in a large series of pediatric patients. Our data demonstrated that percutaneous closure with a PLLA device could be performed with a high short-term success rate and a low complication rate. However, it is associated with a high risk of residual leaks, suggesting that the long-term efficacy of this device may be inadequate for percutaneous ASD closure in pediatric patients, particularly in those with large ASDs.

### 4.1 Device efficacy

Closure success rate of the PLLA device was observed in 96% at 6 months, in 84% at 3 years, and in 88% at 5 years in our study. In the study regarding ASD closure with CBSO, closure success rate was 100% at 2 years (7). In BEST (BioSTAR Evaluation Study), moderate to large shunts on contrast TTE were considered clinically significant, and 96% (54/56) closure success rate was seen at 6 months (4). In a recent study, Ouyang W et al. (15) reported that a novel fully biodegradable ASD occluder (Shanghai Shape Memory Alloy Ltd), constructed from polydioxanone(PDO) and PLA, demonstrated noninferior efficacy and safety compared to a metallic occluder over a 2-year follow-up period. However, nearly half of the cohort consisted of adult patients, a factor that may have under-represented the risk of residual shunts. Our findings indicate that while the short-term efficacy of PLLA-based device was satisfactory, its long-term efficacy in pediatric patients was suboptimal.

Prior studies have indicated that the long-term efficacy of the BioSTAR device may be inferior to that of the Amplatzer and CardioSEAL devices (16, 17). This discrepancy may be attributed to the biodegradability of the membrane component in the BioSTAR device. A similar limitation may potentially apply to the PLLA device. Reasons for the high prevalence of residual leaks in our study may be attributed to the design of the device, and a potential mismatch between the high basal metabolic rates of pediatric patients and the biodegradation profile of the PLLA materials. The self-centering capability of the PLLA occluder is inferior to that of nitinol-based devices. Although a locking mechanism is employed to configure the PLLA framework, the two PLA discs may still fail to maintain secure apposition against the atrial septum in patients with thin septal tissue or floppy rims, which can lead to incomplete endothelialization and procedural failure.

Furthermore, our findings indicate that patients with larger defects, specifically those with a ASD indexed diameter exceeding 15.18 mm/m^2^, exhibited a significantly increased risk of residual leaks, which aligns with previous reports (18). This association may be attributed to the tendency of such patients to present with lower body weight and a thinner interatrial septum (19–21). In these cases, the PLLA device may not generate sufficient compressive force against the septal tissue, potentially contributing to residual leaks, particularly following device degradation. Moreover, ROC analysis also identified the device-to-defect ratio as a significant predictor of residual leak, with an optimal cutoff value of <1.47, suggesting that a smaller device-to-defect ratio may predispose patients to residual shunts. This finding implies that the oversizing strategy may be preferable in this patient population to reduce the risk of residual shunts.

Additionally, pediatric patients have a higher basal metabolic rate compared with adults, which may accelerate the degradation of the PLLA *in vivo*. Based on our follow-up results, the incidence of residual leaks was significantly higher at 2 years post-procedure than at 1 year, with a progressive increase observed over extended follow-up. Notably, the period between 1 and 2 years post-implantation coincides with the onset of systematic degradation of the PLLA occluder. As device degradation advanced, the occurrence of residual leaks increased correspondingly. Therefore, we propose that residual leaks are primarily attributable to the degradation process of the occluder, rather than to defect recanalization.

One of the drawbacks of our study was that our enrollment criteria did not include a retro-aortic rim ≥5 mm as a prerequisite for inclusion, nor was a deficient retro-aortic rim(<5 mm) considered an exclusion criterion. However, during follow-up, we observed that all residual leaks were located at the anterosuperior aspect of the atrial septum, potentially attributable to an insufficient aortic rim, a factor not systematically assessed prior to device implantation, and thus possibly contributing to closure failure. Although aortic rim deficiency has not been demonstrated to be associated with procedural failure in ASD closure using permanent devices (22, 23), its impact remains uncertain when employing totally biodegradable devices. In patients with deficient aortic rims, the PLLA device may not adequately conform to the contour of the aortic wall, potentially resulting in incomplete tissue healing and the development of residual shunts. Based on our findings, the PLLA device may achieve satisfactory closure outcomes in small-to-moderate sized, regularly shaped defects with adequate surrounding rims. However, it may not be suitable for larger defects, particularly those with deficient rims or irregular morphologies.

### 4.2 Device safety

In our study, the complication rate at the 5-year follow-up after PLLA device implantation was 14.3%, consistent with previously reported outcomes for other biodegradable atrial septal implants (4, 7). Specifically, the BEST trial documented a short-term complication rate of 10.7% (4), and a study evaluating the CBSO for interatrial shunt closure reported a comparable rate of 13.3% (7). No major adverse events were observed in our cohort throughout the 5-year evaluation period. In contrast, the pivotal study of the Amplatzer septal occluder reported major complication and overall adverse event rates of 1.6% and 7.2% at 12 months, respectively (24). While the continued access study of the Helex septal occluder documented a 3.6% rate of major adverse events over 5 years (13). Compared with conventional metallic devices, the PLLA device was associated with a lower risk of major complications, a finding that aligns with trial results reported for the PDO/PLA ASD occluder (15).

The most common complication associated with PLLA device closure was cardiac arrhythmia (3 events, 8,6%), which was consistent with published literature identifying arrhythmia as the most frequent complication following ASD closure (25, 26). Notably, one patient developed new-onset moderate MR shortly after implantation of a 30-mm PLLA device, a complication potentially attributable to the device oversizing strategy adopted for large ASDs in this study.

PLLA-based scaffolds have been widely utilized in tissue repair and have demonstrated excellent biocompatibility on the human body (27, 28). Our study further confirms that the compliant mechanical properties of PLLA minimize structural damage to cardiac tissue, as evidenced by the absence of reported cases of cardiac erosion, high-degree atrioventricular block, or severe valvular regurgitation. Nevertheless, the hydrolysis of PLLA generates acidic degradation products, which may pose an increased risk of severe inflammation, thrombosis, and impaired cell differentiation (29). In this study, no evidence of systemic inflammation or thromboembolic events attributable to PLLA degradation was observed, suggesting that PLLA can undergo safe degradation within the cardiac environment. However, no dedicated assessments were conducted to systematically evaluate the potential for microembolization. Therefore, the incidence and clinical significance of thromboembolic events associated with PLLA-based devices in ASD closure remain to be elucidated.

### 4.3 Study limitations

Our study has several limitations. First, the investigation was conducted as a single-center study with a small sample size. Second, the design was single-arm rather than a randomized controlled trial, which introduces inherent limitations in controlling for bias. Third, the predominant reliance on 2D and color-Doppler TTE may have led to underestimation of ASD dimensions and residual shunts. Finally, the detailed biodegradation process of the device was not systematically evaluated in the present study.

## 5 Conclusion

Percutaneous ASD closure using the PLLA device demonstrated an acceptable safety profile in pediatric patients, with a cumulative complication rate of 14.2% and no major adverse events reported over 5 years. However, a relatively high incidence of residual leaks suggests that long-term efficacy may be suboptimal, particularly in patients with larger ASDs. Further long-term follow-up is warranted to more comprehensively evaluate the efficacy and safety of the PLLA device.

## Data Availability

The raw data supporting the conclusions of this article will be made available by the authors, without undue reservation.
