# Supplementary figures and images for: Long-term follow-up results of percutaneous closure of atrial septal defect with a novel biodegradable poly-L-lactic acid device in pediatrics: data from a prospective, single-center trial
Source: Front Cardiovasc Med. 2026 Apr 20;13:1804642. doi: 10.3389/fcvm.2026.1804642 (PMC13147298; doi:10.3389/fcvm.2026.1804642)

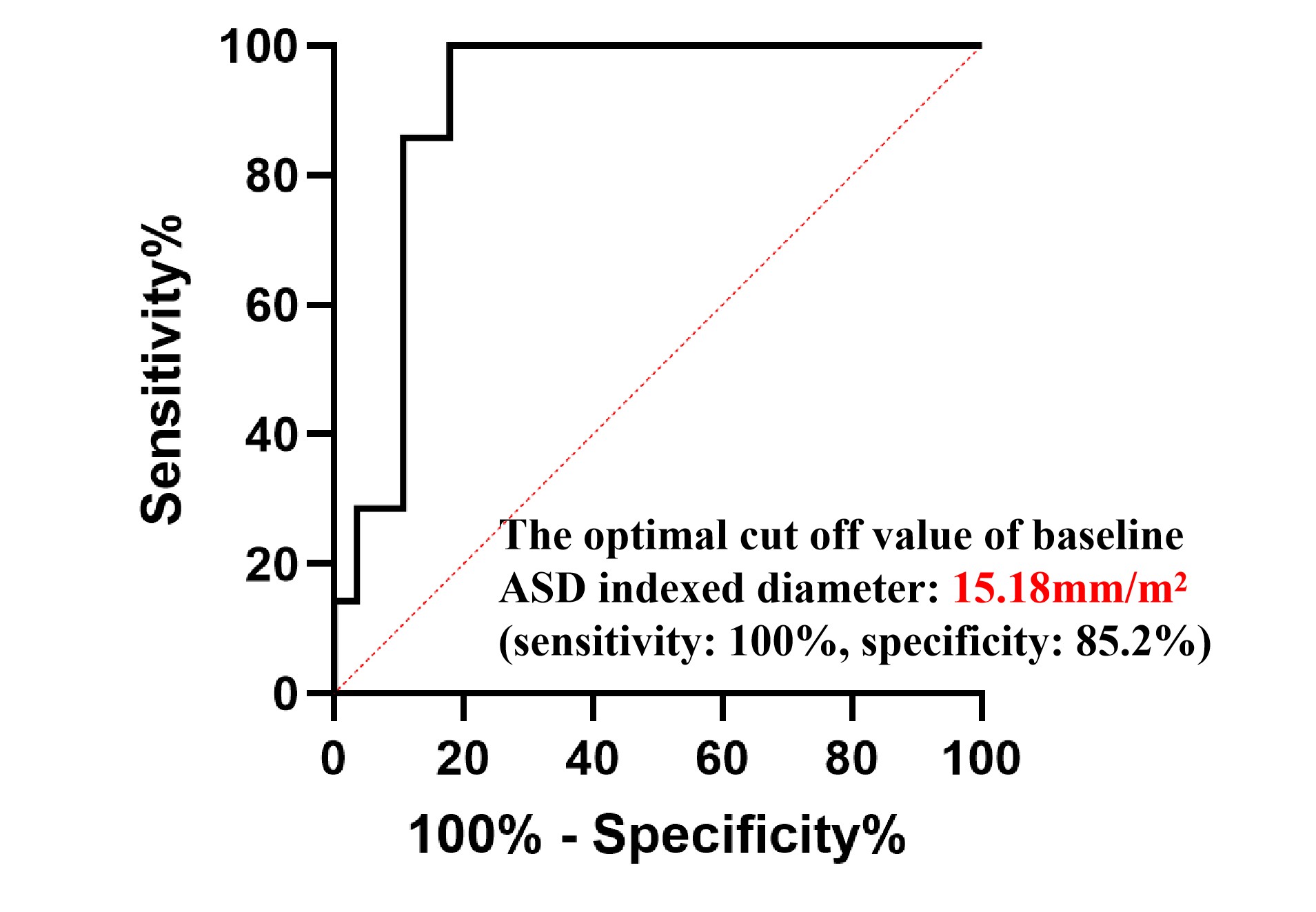

Supplement: Supplementary file 1 [file Image1.jpeg]

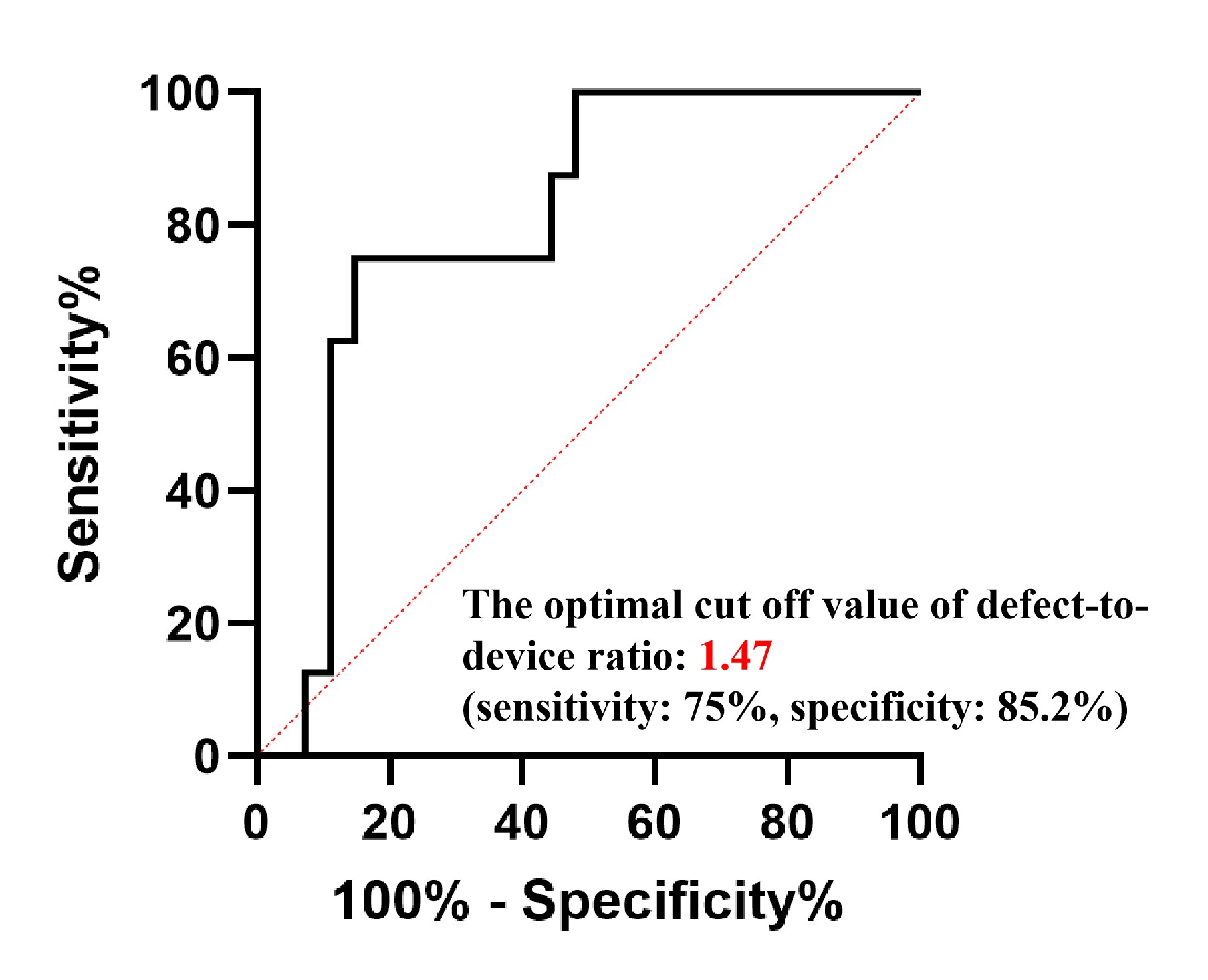

Supplement: Supplementary file 2 [file Image2.jpeg]
